# Evaluation of Hyaluronic Acid Dilutions at Different Concentrations Using a Quartz Crystal Resonator (QCR) for the Potential Diagnosis of Arthritic Diseases

**DOI:** 10.3390/s16111959

**Published:** 2016-11-22

**Authors:** Luis Armando Carvajal Ahumada, Marco Xavier Rivera González, Oscar Leonardo Herrera Sandoval, José Javier Serrano Olmedo

**Affiliations:** 1 Center for Biomedical Technology (CTB), Technical University of Madrid (UPM), 28040 Madrid, Spain; marco.rivera.gonzalez@alumnos.upm.es (M.X.R.G.); josejavier.serrano@ctb.upm.es (J.J.S.O.); 2 Networking Research Center of Bioengineering, Biomaterials and Nanomedicine (CIBER-BBN), Technical University of Madrid (UPM), 28040 Madrid, Spain; 3 Research and Technological Development Center of Electrical, Electronic and ICT Industry (CIDEI), 111311 Bogotá, Colombia; oherreras@ucentral.edu.co; 4 Faculty of Engineering and Basic Sciences, Central University, 111711 Bogotá, Colombia

**Keywords:** viscosity, non-Newtonian fluid, synovial fluid, QCR, arthritic disease

## Abstract

The main objective of this article is to demonstrate through experimental means the capacity of the quartz crystal resonator (QCR) to characterize biological samples of aqueous dilutions of hyaluronic acid according to their viscosity and how this capacity may be useful in the potential diagnosis of arthritic diseases. The synovial fluid is viscous due to the presence of hyaluronic acid, synthesized by synovial lining cells (type B), and secreted into the synovial fluid thus making the fluid viscous. In consequence, aqueous dilutions of hyaluronic acid may be used as samples to emulate the synovial fluid. Due to the viscoelastic and pseudo-plastic behavior of hyaluronic acid, it is necessary to use the Rouse model in order to obtain viscosity values comparable with viscometer measures. A Fungilab viscometer (rheometer) was used to obtain reference measures of the viscosity in each sample in order to compare them with the QCR prototype measures.

## 1. Introduction

The synovial fluid is produced by the synovium membrane inside the articular cartilage. It can be considered a dialysate of plasma with a high content of hyaluronic acid [1,2,3,4]. The amount of the synovial fluid in a joint is no more than 3.5 mL (knee) [4,5] where the main functions of the synovial fluid are the lubrication of the joints and the absorption of the mechanical load produced when walking or running (knees) or holding up weight (cubit and shoulder) [1,2,3,4,5]. The viscosity of the synovial fluid depends on the concentration of the hyaluronic acid. There are studies that relate the low viscosity of the synovial fluid (low concentration of hyaluronic acid) with arthrocentesis, osteoarthritis and inflammatory arthropathies [1,2,3,4,6,7]. Due to the concentration of hyaluronic acid in the synovial fluid being ≈ 3.5 mg/mL [6], the sample may be considered a non-Newtonian fluid with viscoelastic properties [5,8]. Due to this lubricant function, the analysis of synovial fluid is important for the detection and diagnosis of arthritic diseases.

At present, the evaluation of the viscosity of synovial fluid samples is done through macroscopic analysis. This analysis evaluates the color, transparency and viscosity of the sample [1,2,3,9,10]. The results of a macroscopic analysis make the classification of the samples possible in healthy, inflammatory or septic, according to Table 1 [5,9,11].

The viscosity test involves extending the sample on a surface and measuring the length before the thread is cut. If the resulting length is greater than 3 cm, the sample is healthy; if not, the sample displays a problem and more tests are necessary [5].

Although the viscosity of the sample is a fundamental parameter in the macroscopic analysis [3], the clinical method to obtain its value is primarily qualitative. The sample of synovial fluid is obtained through a puncture in the joint and it is extended on a surface with the same needle used to extract it. If the final length is greater than 3 cm, the sample is considered healthy; but if it is lower than 3 cm, the sample is considered abnormal [11].

The macroscopic method provides a subjective measure of viscosity that depends, for example, on the skills and strength of the laboratory researcher, the stretching and the surface. Sometimes the viscosity is not even taken into account, only the appearance [1].

Due to the importance of the hyaluronic acid concentration in the synovial fluid, the small amount of sample in the joints and the current subjective method to obtain a measure of the viscosity, it has been proposed to use a piezoelectric biosensor based on a quartz crystal resonator (QCR) to obtain the measure of viscosity of the sample.

There are several studies linking the use of the Quartz Crystal Microbalance (QCM) to the detection of diseases characterizing biological fluids as blood [12,13,14,15], urine [16,17], saliva [17,18,19] and synovial fluid [20,21]. The methods proposed in these studies have proven to be successful according to their results. However, they are also more complex because they involve the functionalization of the electrode in the crystal in order to detect a specific protein related to the disease. There are also studies that relate similar piezoelectric techniques to sense the viscosity of biological samples, such as: the use of cantilevers [22,23,24]. Sensors based on cantilevers are also valid to obtain the viscosity values in biological samples; the physics principle is the same. However, the cantilever transducer is more fragile because it only has one fixed contact point. In consequence, the manufacture and use of these biosensors have a higher level of difficulty.

In contrast to the standard method applied in clinical and others more complex piezoelectric techniques, the method proposed enables us to know the viscosity value for synovial fluid samples in a simple way because the proposed technique does not require the functionalization of the electrode and the manufacture of the biosensor is easy.

In preliminary tests, aqueous dilutions of hyaluronic acid at different concentrations have been used to emulate the synovial fluid (both healthy and abnormal). As reference equipment, the Fungilab viscometer has been used to obtain viscosity measures of each sample in order to compare them with experimental measures.

## 2. Theory

The present theory is exposed in two parts. The first involves the design and manufacture of the piezoelectric biosensor, and the second contains the mathematical explanation about the viscoelastic behavior of the samples used and the equations needed to make the proposed analysis.

### 2.1. Quartz Crystal Resonator

The quartz crystal resonators are electro-mechanical transducers with two electrodes which are sensitive to the deposition of mass [25,26,27,28] or to contact with liquid [29,30,31]. The quartz crystal has a resonance frequency determined by its geometry, in particular, by its thickness. The crystal is stimulated by an AC voltage between its electrodes and, as a consequence, the crystal vibrates producing shear waves [32]. When the signal frequency is close to the resonance frequency of the crystal, the intensity of the vibration increases. By performing a frequency sweep close to the resonance, and measuring the voltage and current in the crystal, it is possible to know the behavior of the admittance of the crystal for such frequencies [30,31,33,34,35,36].

When the crystal is in contact with a mass or liquid, its resonance frequency changes according to two equations: the Sauerbrey equation for mass deposition (Equation (1)) [25] and the Kanazawa equation (Equation (2)) for fluids [37]:

(1) $$\Delta f=-\frac{2{f_{0}}^{2}}{A\sqrt{\rho_{q}G_{q}}}\Delta m$$

(2) $$\Delta f=-\sqrt{n}{f_{0}}^{3/2}\sqrt{\frac{\rho_{L}\eta_{L}}{\pi \rho_{q}G_{q}}}$$

where $\rho_{q}$ and $G_{q}$ are the specific density and the shear modulus of quartz respectively, $f_{0}$ is the fundamental resonance frequency of the quartz, related to its thickness, and Δm is the thin film of mass deposited, A is the piezoelectrically active crystal area, $\rho_{L}$ and $\eta_{L}$ are the density and viscosity of the fluid respectively and n is the overtone number [30,37,38].

The Kanazawa equation only applies to Newtonian liquids (the viscosity is always constant) [34]. However, aqueous dilutions of hyaluronic acid and the synovial fluid are Non-Newtonian fluids [5,39,40]. For this reason, it is necessary to use a more general equation to find the viscosity value:

(3) $$\Delta F=\frac{-1+i}{\sqrt{2}\pi \sqrt{\rho_{q}G_{q}}}\sqrt{\rho_{L}\omega \left(\eta_{L}^{\prime }-i\eta_{L}^{\prime \prime }\right)}$$

(4) $$\eta_{L}^{\prime }=\frac{G_{L}^{\prime \prime }}{\omega }=-\frac{\pi \rho_{q}G_{q}}{\rho_{L}f_{r}}\frac{\Delta F\Delta \Gamma }{f_{0}^{2}}$$

(5) $$\eta_{L}^{\prime \prime }=\frac{G_{L}^{\prime }}{\omega }=\frac{1}{2}\frac{\pi \rho_{q}G_{q}}{\rho_{L}f_{r}}\frac{\left(\Delta \Gamma^{2}-\Delta F^{2}\right)}{f_{0}^{2}}$$

**Figure 1:**
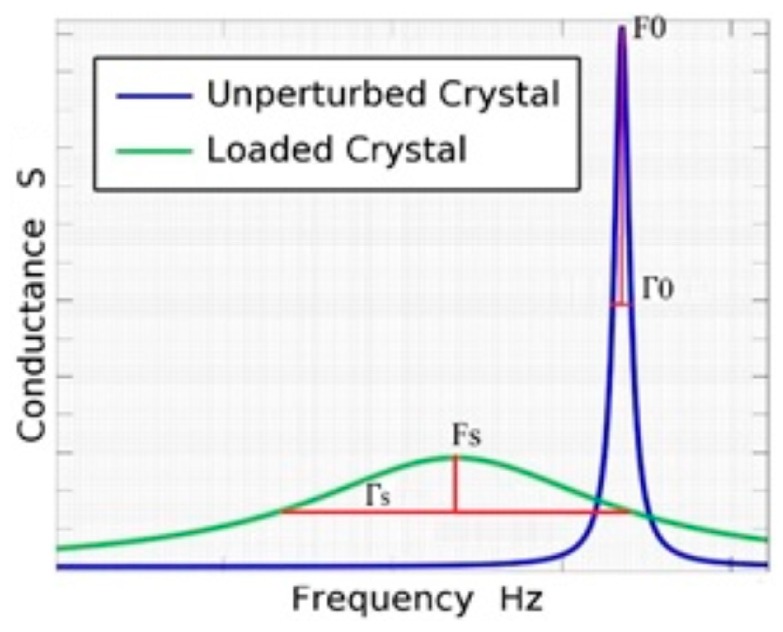
Change in the morphology of the conductance curve for bare crystal and in contact with a liquid sample.

According to Johannsmann [34], Equations (3)–(5) are more general expressions and these can be used to obtain the values of two components of the viscosity for non-Newtonian fluids. ω is the angular frequency, $\eta_{L}^{\prime }$ and $\eta_{L}^{\prime \prime }$ are the real and the imaginary parts of the viscosity, and *G’_L_* and *G’’_L_* are the real and the imaginary part of the shear modulus for the liquid respectively. ΔF and Δ*Γ* are the resonance frequency shift and the bandwidth shift respectively (Figure 1).

According to Figure 1, Δ*f* and Δ*Γ* are obtained by using the following equations:

(6) $$\Delta f=f_{s}-f_{0}$$

(7) $$\Delta \Gamma =\Gamma_{s}-\Gamma_{0}$$

For Newtonian liquids ($\eta_{L}^{\prime }$ = const, $\eta_{L}^{\prime \prime }$ = 0), Δf and Δ*Γ* are equal and opposite. For viscoelastic liquids ($\eta_{L}^{\prime }$ = *η*(*ω*), $\eta_{L}^{\prime \prime }$ ≠ 0) [34].

There is another important variable called motional resistance (*R*). Motional resistance is defined as the impedance value at series resonant frequency [41]. This resistance is related to mechanical energy losses in the crystal during its vibration [31]. In this study, R is calculated as the inverse value of the conductance curve peak. The change in motional resistance (Δ*R*) due to the deposition sample is calculated based on the following equation:

(8) $$\Delta R=\frac{R_{s\left(with\;sample\right)}}{R_{0\left(undamped\right)}}$$

### 2.2. Homogeneous Viscoelastic Samples with Pseudo-Plastic Behavior

When a viscoelastic sample with pseudo-plastic behavior is deposited and characterized using a QCR, the frequency shift obtained is several times lower than expected [39,40]. In consequence, the Equations (3)–(5) cannot be applied directly because the apparent viscosity obtained will be similar to the viscosity of the sample solvent [39].

**Figure 2:**
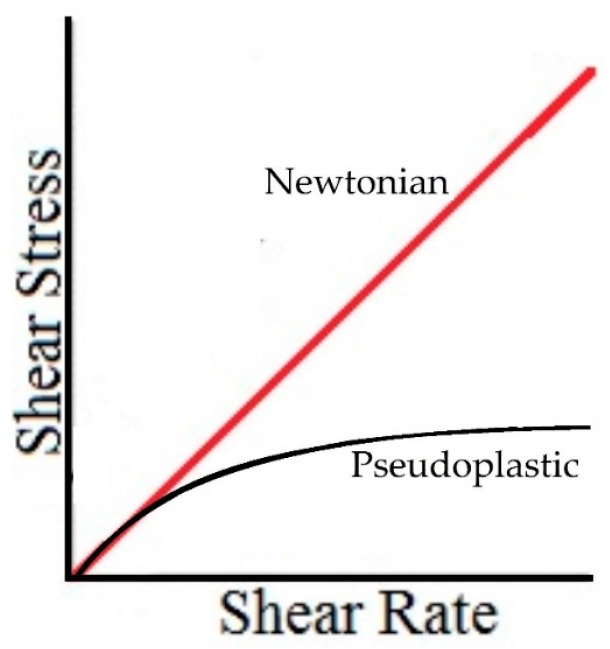
Shear Stress and Shear Rate for Newtonian (red) and Pseudoplastic (black) fluids.

Additionally, it is not possible to compare the QCR measures with the viscometer measures directly because the pseudoplastic behavior is related to the shear rate. In other words, the apparent viscosity for pseudoplastic fluids (hyaluronic acid), defined as the slope of the curve in Figure 2, decreases when the shear rate increases [42]. For Newtonian fluids, it is constant. Figure 2 presents this phenomenon.

**Figure 3:**
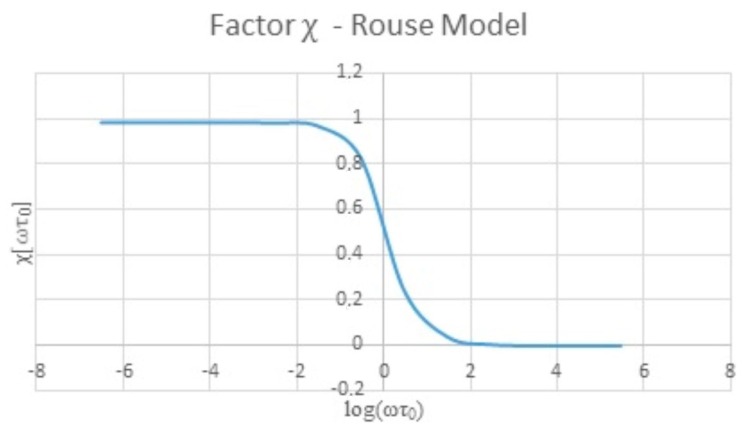
Factor χ for polymeric fluids (pseudoplastic behavior).

The shear rate applied to the viscometer is several units of magnitude lower than the shear rate in the QCR because the frequency in the viscometer is less to 1 Hz and the QCR works at 10 MHz. In order to compare the apparent viscosity at a low shear rate (viscometer) with the apparent viscosity at a high shear rate (QCR), an additional factor χ[ωτ_0_] is necessary, where ω is the angular frequency and τ_0_ is the relaxation time of the sample [40]. According to [39,40], the Rouse model is appropriate to explain the behavior of polymeric fluids like hyaluronic acid samples at different shear rates. Figure 3 shows this behavior.

In the Rouse model, the relaxation time is defined as [39,40,43]:

(9) $$\tau_{0}=\frac{6\eta_{0}M}{\pi^{2}\rho RT}$$

where *M* is the molecular weight of the polymer, *R* is the gas constant (8.314 J·K^−1^·mol^−1^), *T* is the absolute temperature (288 K for experiments), *η*_0_ is the zero shear rate viscosity and *ρ* is the fluid density. Zero shear rate viscosity was obtained using the Fungilab viscometer at the minimum shear rate (0.1 rpm). These values have been saved into a database in the biosensor’s software.

According to [40], the frequency shift Δω obtained can be expressed as:

(10) $$\Delta \omega =\Delta \omega_{0}\cdot \chi \left[\omega \tau_{0}\right]$$

where, Δ*ω*_0_ is only related to the viscous component of the viscoelastic sample (Newtonian behavior). This equation is the same Kanazawa Equation (2):

(11) $$\Delta \omega_{0}\;\sim \;{\omega_{0}}^{-2/3}\sqrt{\left(\frac{\rho \eta_{0}}{\rho_{q}G_{q}}\right)}$$

Using factor *χ* and the frequency shift Δω obtained with the QCR, is possible to obtain a new frequency shift value Δ*ω*_0_ comparable to the viscometer measure. In other words, factor *χ* allows us to obtain the frequency shift generated for an equivalent Newtonian fluid (viscous component) Δ*ω*_0_ by using the QCR measures.

According to [40], factor *χ* is defined as:

(12) $$\chi \left[\omega \tau_{0}\right]=\sqrt{\frac{\eta \prime }{\eta_{0}}}\sqrt{\left(\frac{\sqrt{\left(1+tan^{2}\left(\delta \right)\right)}-1}{\tan\left(\delta \right)}\right)}$$

where the loss tangent $\tan\left(\delta \right)=\eta^{\prime }\left[\omega \right]/\eta^{\prime \prime }\left[\omega \right]$.

Using the generalized Maxwell model [40,44] the following equations are obtained (Appendix A):

(13) $$\frac{\eta \prime }{\eta_{0}}=\frac{1}{\zeta \left(\nu \right)}\overset{\infty }{\underset{k=1}{\sum }}\frac{k^{\nu }}{k^{2\nu }+{\left(\tau_{0}\omega \right)}^{2}}$$

(14) $$\frac{\eta \prime \prime }{\eta_{0}}=\frac{\tau_{0}\omega }{\zeta \left(\nu \right)}\overset{\infty }{\underset{k=1}{\sum }}\frac{k^{\nu }}{k^{2\nu }+{\left(\tau_{0}\omega \right)}^{2}}$$

For the Rouse model, *ν* = 2 and *ζ*(*ν*) is the Riemann Zeta Function [40,44].

## 3. Materials and Methods 

### 3.1. Samples

The concentration of the hyaluronic acid in the healthy synovial fluid is around 3.5 mg/mL [6,7,45]. In traumatic arthritis and osteoarthritis the hyaluronic acid concentration decreases to around 1.3 mg/mL, and for rheumatoid arthritis the concentration is lower, only 0.84 mg/mL [6]. In this study, the hyaluronic acid dilutions were done at the same concentrations in order to emulate the healthy and abnormal synovial fluid. One additional sample at 7 mg/mL was made with the purpose of obtaining an extra point in the analysis of results. The hyaluronic acid (Mw = 1.5 MDa) was supplied by Sigma Aldrich (St. Louis, MO, USA).

Dilutions were prepared by weighing the hyaluronic acid salt (HA) using the analytic balance and mixing the HA with cold water, (water temperature was 4 °C). The amount of hyaluronic acid and the water volume were calculated according to the desired concentration. After mixing HA and cold water, the solution was stirred gently and continuously during 5 min. Finally, the samples were stored in the refrigerator for 8 h. This process is needed so that the mixture does not contain air bubbles (homogeneous solution). With the aim of evaluating the performance of the QCR biosensor and its detection limit, additional glycerol solutions were created at different concentrations in milli-q water (40%, 30%, 20%, 10%, 5%, 2.5%, 1.25%, 0.63%, 0.31%).

### 3.2. Crystals and Holder Cell

The 10 MHz quartz crystals were purchased from International Crystal Manufacturing (Oklahoma City, OK, USA). The static cell was obtained from Gamry Instruments Inc. (Warminster, PA, USA). 

### 3.3. Viscometer of Reference

The rotational viscometer Alpha series manufactured by Fungilab has been used as reference equipment for the viscosity measures. Its resolution is 0.1 mPa·s according to manufacturer. This equipment is in the Center for Biomedical Technology (CTB).

### 3.4. Biosensor

**Figure 4:**
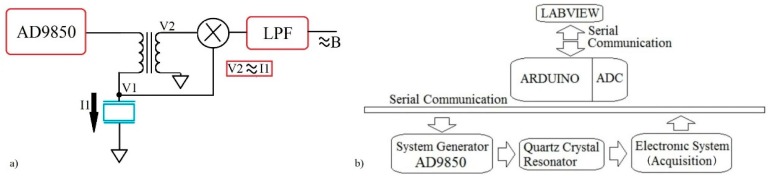
(**a**) Simplified Diagram Acquisition System of the Biosensor; (**b**) Blocks Diagram System of the Biosensor.

The quartz crystal has two electrodes, one on each side. The active electrode receives the sample on its surface and is connected to the electric ground to give more stability to the measures. The proposed circuit gets the voltage and current signal for several frequencies in a configurable bandwidth close to the crystal series resonance frequency (Fs). The circuit is a variation of the Nakamoto and Kobayashi proposal [46]. The electronic system includes a DAC (AD9850 module, Analog Devices, Norwood, MA, USA) to get a high resolution in the measures. The AD9850 module is capable of creating a sinusoidal signal of between 1 and 40 MHz with a frequency step of 1 Hz. The amplitude of the input signal in the crystal is 1 Vp. The sweep frequency is configured close to the resonance frequency of the quartz crystal to identify the expected behavior. The quartz crystal is placed in an array with a toroidal-core transformer used as a current sensor [30,46]. The secondary coil of the transformer gives a voltage proportional to the current in the primary which is the crystal current. The voltage is shifted 90° with respect to the original current signal. The voltage signal (V2) related to the current through the crystal (I1) and the voltage used to stimulate the crystal (V1) are multiplied by using an AD835 mixer (manufacturer) according to Figure 4a.

The output signal in the mixer is filtered using an active filter with an LT1057 amplifier (Linear Technology, Milpitas, CA, USA) to obtain the DC component. According to [30], the DC component is related to the susceptance of the quartz crystal.

The acquisition system uses the ADC port of the Arduino DUE card (https://www.arduino.cc/) to obtain the output signals (voltage, current, susceptance). The digital resolution of the acquisition is 12 bits. With this system, the acquisition time per sweep cycle takes a few seconds. Figure 4b shows the blocks diagram of the prototype.

**Figure 5:**
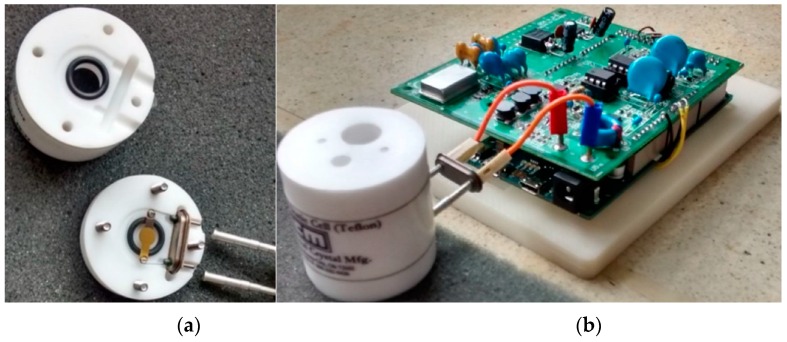
Gamry Cell (**a**) and Prototype System (**b**).

A Labview code has been developed to control the Arduino card. Through this program the user can choose the initial parameters (range of frequency sweep and amplitude of signal) and obtain the voltage and current signals on the quartz crystal for every frequency from it. The conductance and susceptance graphs are shown in the Labview interface, ΔF, ΔΓ and ΔR are calculated with the same code. Figure 5 displays the QCR prototype system.

Additionally, the Labview software has access to a database created with the external variables needed to obtain the correction factor χ: zero shear viscosity, molecular weight, the gas constant, the density of the fluid, temperature and the Riemann Zeta Function value for the Rouse model. The software uses the database in order to calculate the factor χ. The user can edit the zero shear viscosity, the density or the temperature if the value is known.

In this study, the concentration of the samples is always known. However, for an unknown sample, the biosensor uses the change in the motional resistance (ΔR) in order to select the correct factor χ. In the results section is shown the ΔR values for each sample. These resistance values are also saved in the database for reference.

### 3.5. Measurement Protocol

(1) Select a frequency range to make the sweep and to obtain the conductance curve in the frequency domain for the bare crystal (without sample). Calculate the serial resonance frequency (*F*_0_), the half band half width (*Γ*_0_) and the motional resistance (*R*_0_) using the developed code in Labview for each cycle. These measures are taken 10 times and the final values are the average of the measures; (2) Have the sample analyzed in the electrode surface of the quartz crystal (50 μL of volume); (3) Adjust the frequency range in Labview software to obtain the conductance curve of the quartz crystal in contact with liquid sample; (4) The measurements with samples (*F*_s_, *Γ*_s_ and *R*_s_) are taken 100 times (approx. 3 min) and the final values are the average among the measures; (5) Obtain ΔF, ΔΓ and ΔR.

The density values were obtained using an analytical balance. The mass of each solution was determined from 1 mL of its volume.

### 3.6. Protocol to Clean the Crystal

(1) Add abundant water to wash the residues on the crystal; (2) Add alcohol (isopropanol) to clean the crystal surface; (3) Add acetone to complete the electrode cleaning (remove solvents); (4) Dry the electrode surface with a liquid nitrogen pistol.

## 4. Results

### 4.1. Biosensor Performance

The sensitivity of the QCR prototype was obtained by following the measurement protocol steps using glycerol aqueous solutions at concentrations commented in Section 3.1.

Table 2 shows the viscosity obtained using the Kanazawa Equation (2). The results were obtained by averaging twenty individual measures for each solution. Variability in measures is in part due to the cleaning process and the crystal reposition into the cell.

**Figure 6:**
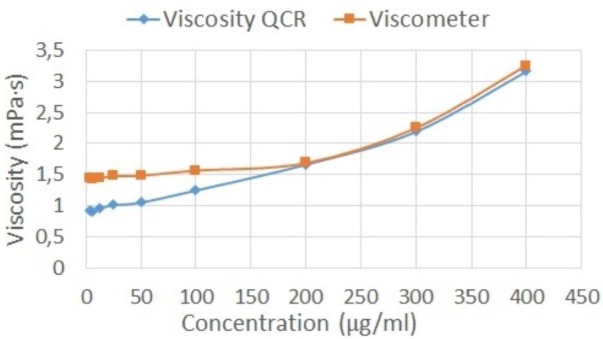
QCR biosensor performance vs. Fungilab viscometer performance.

The results allow us to see the capacity of the prototype sensor to detect the viscosity samples in up to 6.3 mg/mL of the solution (limit of detection). Figure 6 shows the performance of the QCR biosensor vs. Fungilab Viscometer. According to the Figure 6, the Fungilab viscometer does not differentiate samples with a concentration lower than 5%.

**Figure 7:**
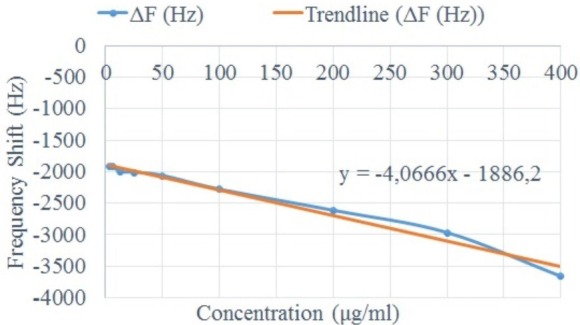
Response curve—Δf vs. Concentration of the glycerol samples (Newtonian behavior).

According to the response curve (Figure 7), the sensitivity of the QCR prototype is around 4 Hz per 1 μg/mL of sample concentration change.

### 4.2. Hyaluronic Acid Samples

According to the methods exposed, the following data were obtained for the hyaluronic acid mixtures:

Table 3 shows the measures obtained using the QCR prototype with different samples deposited on the electrode crystal. F0, ΔF, ΔΓ and ΔR correspond to the average of 20 independent measures for each sample.

Table 4 shows the apparent viscosity obtained using the Equations (4)–(6) with the frequency shift Δf and bandwidth shift ΔΓ measured. It is not possible to compare at a high shear rate (QCR measures) and low shear rate (viscometer measures) for the same sample.

According to the methods section, it is necessary to use factor χ to compare the biosensor measures with the viscometer measures. Using Equations (9)–(14), factor χ[ωτ_0_] and the frequency shift Δω0 solely related to the viscous component of the viscoelastic sample were calculated for each sample. Table 5 and Table 6 show the results.

**Figure 8:**
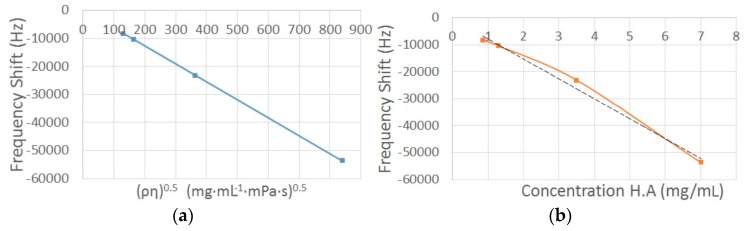
Response curve of the QCR biosensor vs. the square root of the density-viscosity product (**a**) and Response curve of the QCR biosensor vs. concentration (**b**).

Figure 8 shows the response curve of the biosensor versus the square root of the density viscosity product (Figure 8a) and versus the concentration of hyaluronic acid in the samples (Figure 8b). The frequency shift shown corresponds only to the viscous component of the viscoelastic samples.

**Figure 9:**
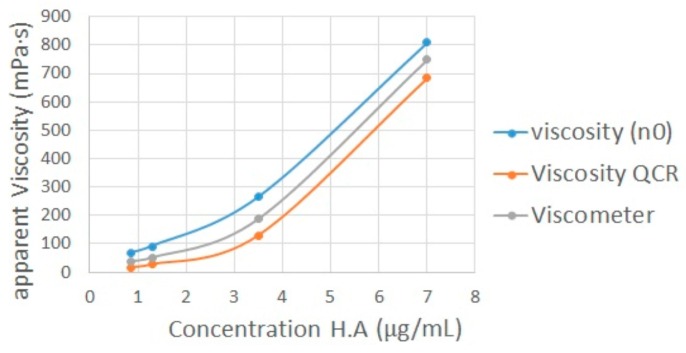
Apparent viscosity obtained by Rouse model vs. viscometer measures.

Figure 9 shows the comparison between the apparent viscosity obtained by Rouse model using the QCR measures and the viscometer measures. The grey data in Figure 9 were obtained with the same Fungilab viscometer at a higher shear rate (1.5–2.5 rpm) than zero shear rate viscosity (0.1 rpm).

## 5. Discussion

The proposed QCR prototype has a good performance. The results illustrate the capability of the QCR sensor to measure small changes in the composition of glycerol aqueous solutions. Figure 7 shows the relationship between the concentration of the sample and the sensor response ΔF. According to Figure 7, the sensitivity of the sensor is ≈ 4.06 Hz per 1 μg/mL concentration sample. The smallest incremental change of concentration that could be detected in the biosensor (resolution) was 6.25 μg/mL.

According to the results for hyaluronic acids solutions, the presence of hyaluronic acid in an aqueous solution results in a complex rheological behavior; this solution is catalogued into the pseudoplastic fluids. Due to this viscoelastic and pseudoplastic behavior, it is not possible to compare the QCR measures (high shear rate) and the viscometer measures (low shear rate) for the same sample. Table 4 shows these results.

The QCR measures require an additional factor to obtain results comparable with the viscometer results. The Rouse model is a good alternative to obtain the additional factor χ in order to correct the experimental measures.

By applying the Rouse model to the experimental results, the new apparent viscosities were obtained for each sample. These apparent viscosities are only related to the viscous component of hyaluronic acid solutions. In consequence, the new apparent viscosities exhibit the same behavior as the viscometer measures (Figure 9). In other words, the new frequency shifts obtained correspond to an equivalent fluid with the viscous component alone. The new apparent viscosities obtained were calculated using Equation (11). Table 6 shows the results obtained after applying the Rouse model. The relative error increases as the concentration of the sample decreases. However, the viscosities obtained after applying the Rouse model are comparable with the reference viscosities obtained with the viscometer.

In this study, the concentration of the hyaluronic acid is already known for every sample. However, for future clinical samples, the concentration, the zero shear viscosity and the density are unknown variables. In such cases, it is necessary to use the motional resistance value in order to relate the response of the QCR with the correction factor χ. For unknown samples, the ΔR value obtained experimentally is compared with the ΔR values in the database and the system selects the parameters required to calculate the factor χ and adjust the viscosity value. Table 3 shows the ΔR values obtained for each sample.

The obtained results confirm the relationship between hyaluronic acid concentration and the viscosity of the fluid. Considering the concentration samples used in this study are very similar to the ones reported for arthritic diseases [6,7] and the results shown, the QCR biosensor would be capable of identifying synovial fluid samples (healthy or with any medical affectation).

## 6. Conclusions

Quartz crystal resonators are capable of identifying changes of viscosity in biologic fluid samples due to changes in the concentration of the fluid. The sensitivity of the QCR prototype was around 4 Hz per 1 μg/mL of sample concentration change. In this study, aqueous solutions of hyaluronic acid at different concentrations have been characterized through a QCR biosensor. The results prove that it is possible to relate the concentration of hyaluronic acid with the sample viscosity. Considering that hyaluronic acid is a key component in synovial fluid and taking into account that the hyaluronic acid concentrations used in this work are related to arthritic diseases [6,7], the QCR biosensor may be used in applications related to the diagnosis of such diseases. Additionally, the QCR technique may be beneficial in synovial fluid characterization because the amount of synovial fluid available in clinical samples is too little. For physicians, the fact of knowing the viscosity value of the synovial fluid in their patients will help them to make a timelier diagnosis, and to prepare more specific treatments in accordance with the status of the disease. As future work, testing will be performed with real synovial fluid in small mammals. Also, an artificial neural network will be implemented with a robust database. Such a neural network will provide the sensor a better capacity to select the correct factor χ to adjust the viscosity value.

## Figures and Tables

**Table 1:** Macroscopic classification of synovial samples.

	Normal	Inflammatory	Septic	Hemorrhagic
Appearance	Transparent	Turbid	Turbid	Bloody
Color	Straw-colored	Yellow-cloudy	Opaque	Red-opaque
Viscosity	High	Low	Low	Variable

**Table 2:** Glycerol solutions results (average ± SD).

Concentration (μg/mL)	ΔFs (Hz)	ΔΓ (Hz)	Density (mg/mL)	Viscosity (mPa·s)
400.0	−3660 ± 49	3697 ± 23	1106	3.1679 ± 0.08
300.0	−2968 ± 51	3042 ± 12	1054	2.1935 ± 0.07
200.0	−2617 ± 86	2619 ± 14	1048	1.6643 ± 0.05
100.0	−2281 ± 48	2287 ± 33	1023	1.2470 ± 0.01
50.0	−2064 ± 32	2076 ± 21	1010	1.0525 ± 0.03
25.0	−2011 ± 22	2032 ± 28	999	1.0097 ± 0.03
12.5	−2004 ± 54	2033 ± 33	1007	0.9544 ± 0.01
6.3	−1920 ± 49	1926 ± 21	1006	0.9020 ± 0.01
3.1	−1911 ± 92	1943 ± 18	998	0.9140 ± 0.06

**Table 3:** Hyaluronic Acid (H.A) results (Average ± SD).

Concentration H.A (mg/mL)	F_0_ (Hz)	ΔF (Hz)	ΔΓ (Hz)	ΔR	Density (mg/mL)	Viscosity (η0) (mPa·s)
7.00	9983452 ± 45	−3411 ± 11	3615 ± 15	24.6 ± 0.42	1032.23	809
3.50	9983394 ± 54	−3381 ± 12	3563 ± 13	21.1 ± 0.48	1017.89	265
1.29	9983373 ± 33	−3315 ± 8	3463 ± 15	18.5 ± 0.52	1014.32	91
0.85	9983428 ± 44	−3269 ± 6	3341 ± 9	16.2 ± 0.36	1019.53	68

**Table 4:** Apparent viscosity obtained with the QCR biosensor and Fungilab viscometer.

Concentration H.A (mg/mL)	QCR-Viscosity (mPa·s)	Viscometer (mPa·s)
7	3.25	748.12
3.5	3.13	186.60
1.29	2.99	50.00
0.85	2.85	37.78

**Table 5:** Factor χ.

Concentration H.A (mg/mL)	τ (μs)	η′/η_0_	η″/η_0_	tan(δ)	χ
7.00	0.30	0.15	2.84	0.05	0.06
3.50	0.10	0.27	1.66	0.16	0.15
1.29	0.04	0.46	0.99	0.46	0.32
0.85	0.03	0.54	0.85	0.63	0.39

**Table 6:** ΔF_0_ and equivalent viscosity related only to the viscous component of hyaluronic acid mixtures.

Concentration H.A (mg/mL)	ΔF_0_ (Hz)	Equivalent Viscosity QCR (mPa·s)	Viscometer (mPa·s)	% Error with Viscometer
7.00	−53603	683.6	748.12	9%
3.50	−23175	129.6	186.60	31%
1.29	−10476	26.8	50.00	46%
0.85	−8323	16.7	37.78	56%

## Abbreviations

The following abbreviations were used in this manuscript:

H.A	Hyaluronic Acid
QCR	Quartz crystal resonator
M	Media
SD	Standard deviation

## Appendix A

Equations (12) and (13) emerge from the Maxwell constitutive Equation [44]:

(A1) $$\tau +\lambda_{1}\frac{\partial \tau }{\partial t}=-\eta_{0}\dot{\gamma }$$

where: $\dot{\gamma }$ is the rate of strain tensor, *η*_0_ is the zero shear rate viscosity and $\lambda_{1}$ is the relaxation time.

Solving for *τ*, integrating:

(A2) τ(t)=−∫−∞t(η0λ1γ˙(t′))et′λ1dt′etλ1+ke−tλ1

where primes have been added to *t*’s in the integrand in order to avoid confusion. k is the integration constant. If we prescribe that the stress in the fluid is finite at *t* = −∞, we must set *k* on zero.

Applying L’Hopital’s rules in Equation (A2):

(A3) limt→−∞τ(t)=limt→−∞(−(η0λ1γ˙(t))etλ11λ1etλ1)=−η0γ˙(−∞)

Therefore, if $\dot{\gamma }\left(-\infty \right)$ is finite, the stress is also finite at *t* = −∞. Consequently, the Maxwell constitutive equation can be written in the form:

(A4) τ(t)=−∫−∞t(η0λ1e−(t−t′)λ1)γ˙(t′)dt′

The quantity in brackets is called the relaxation modulus for Maxwell fluid. This expression shows that the stress at the present time *t* depends on the rate of strain at time *t* as well as on the rate of strain at all past times *t*’ with a weighting factor (relaxation modulus) that decays exponentially as one goes backwards in time.

Taking into account:

(A5) $$\gamma_{yx}\left(t_{0},t\right)=\int_{t_{0}}^{t}{\dot{\gamma }}_{yx}\left(t\prime \right)dt\prime$$

Then:

(A6) $$\frac{\partial \gamma }{\partial t\prime }=\dot{\gamma }\left(t\right)$$

(A7) $$\gamma_{yx}\left(t,t\prime \right)=\int_{t}^{t\prime }\dot{\gamma }\left(t\prime \prime \right)dt\prime \prime$$

Now, integrating by parts *τ*(*t*):

(A8) τ(t)=+∫−∞t(η0λ12e−(t−t′)λ1)γ˙(t,t′)dt′

The quantity in brackets is called memory function for Maxwell fluid. In this form the Maxwell model states that the stress at the present time *t* depends on the history of the strain for all past times −∞<t′<t.

Now, considering the Equation (1) as a sums of partial stresses (Generalized Maxwell Model):

(A9) $$\tau \left(t\right)=\overset{\infty }{\underset{k=1}{\sum }}\tau_{k}\left(t\right)$$

(A10) $$\tau_{k}+\lambda_{k}\frac{\partial \tau_{k}}{\partial t}=-\eta_{k}\dot{\gamma }$$

The shear stress can be written as:

(A11) τ(t)=−∫−∞t[∑k=1∞ηkλke−(t−t′)λk]γ˙(t′)dt′

(A12) τ(t)=+∫−∞t[∑k=1∞ηkλk2e−(t−t′)λk]γ˙(t′)dt′

where:

(A13) $$\eta_{k}=\frac{\lambda_{k}}{\sum_{k}\lambda_{k}}$$

(A14) $$\lambda_{k}=\frac{\lambda }{k^{\alpha }}$$

λ is a constant time and *α* is a dimensionless quantity which describes the slope of $\eta^{\prime }$ versus *ω* on a log-log plot at high *ω*.

The Rouse molecular theory for dilute polymer solutions gives *α* = 2. Additionally, in the general linear viscoelastic model, the Equations (A11) and (A12) can be written as:

(A15) $$\tau =-\int_{-\infty }^{t}G\left(t-t\prime \right)\dot{\gamma }\left(t\prime \right)dt\prime$$

(A16) $$\tau =+\int_{-\infty }^{t}M\left(t-t\prime \right)\dot{\gamma }\left(t,t\prime \right)dt\prime$$

where the memory function *M* is the derivative of the relaxation modulus *G*. Now, supposing a small amplitude oscillatory motion for the fluid:

(A17) $${\dot{\gamma }}_{yx}\left(t\right)={{\dot{\gamma }}^{0}}_{yx}\cos\left(\omega t\right)$$

${{\dot{\gamma }}^{0}}_{yx}$ is real and positive.

Resolving Equation (A15) taking into account Equation (A17):

(A18) $$\tau_{yx}=-\int_{-\infty }^{t}G\left(t-t^{\prime }\right){{\dot{\gamma }}^{0}}_{yx}\cos\left(\omega t^{\prime }\right)dt^{\prime }$$

(A19) $$\tau_{yx}=-{{\dot{\gamma }}^{0}}_{yx}\int_{0}^{\infty }G\left(s\right)\cos\left(\omega \left(t-s\right)\right)ds$$

(A20) $$\tau_{yx}=-\left[\int_{0}^{\infty }G\left(s\right)\cos\left(\omega s\right)ds\right]{{\dot{\gamma }}^{0}}_{yx}\cos\left(\omega t\right)-\left[\int_{0}^{\infty }G\left(s\right)\sin\left(\omega s\right)ds\right]{{\dot{\gamma }}^{0}}_{yx}\sin\left(\omega t\right)$$

Considering:

(A21) $$\tau_{yx}=-\eta \prime {{\dot{\gamma }}^{0}}_{yx}\cos\left(\omega t\right)-\eta \prime \prime {{\dot{\gamma }}^{0}}_{yx}\sin\left(\omega t\right)$$

Then:

(A22) $$\eta^{\prime }=\int_{0}^{\infty }G\left(s\right)\cos\left(\omega s\right)ds$$

(A23) $$\eta^{\prime \prime }=\int_{0}^{\infty }G\left(s\right)\sin\left(\omega s\right)ds$$

Now, replacing the Equation (A11) in Equation (A22):

(A24) η′=∫0∞[∑k=1∞ηkλke−(s)λk]cos(ωs)ds

Solving for integration by parts two times:

(A25) ∫0∞[∑k=1∞ηkλke−(s)λk]cos(ωs)ds = ∑k=1∞ηkλk21ω2−∑k=1∞(1λk2ω2)*[∫0∞[∑k=1∞ηkλke−(s)λk]cos(ωs)ds]

This equation can be written as:

(A26) $$\eta^{\prime }=\overset{\infty }{\underset{k=1}{\sum }}\frac{\eta_{k}}{{\lambda_{k}}^{2}}\frac{1}{\omega^{2}}-\overset{\infty }{\underset{k=1}{\sum }}\left(\frac{1}{{\lambda_{k}}^{2}\omega^{2}}\right)*\eta^{\prime }$$

Solving for $\eta^{\prime }$:

(A27) $$\eta^{\prime }=\overset{\infty }{\underset{k=1}{\sum }}\frac{\eta_{k}}{1+{\left(\lambda_{k}\omega \right)}^{2}}$$

Solving in similar way for $\eta^{\prime }\prime$:

(A28) $$\eta^{\prime \prime }=\overset{\infty }{\underset{k=1}{\sum }}\frac{\eta_{k}\lambda_{k}\omega }{1+{\left(\lambda_{k}\omega \right)}^{2}}$$

In addition, using the expressions (A13) and (A14) in Equations (A27) and (A28), these results become:

(A29) $$\frac{\eta \prime }{\eta_{0}}=\frac{1}{\zeta \left(\alpha \right)}\overset{\infty }{\underset{k=1}{\sum }}\frac{k^{\alpha }}{k^{2\alpha }+{\left(\lambda \omega \right)}^{2}}$$

(A30) $$\frac{\eta \prime \prime }{\eta_{0}}=\frac{\lambda \omega }{\zeta \left(\alpha \right)}\overset{\infty }{\underset{k=1}{\sum }}\frac{k^{\alpha }}{k^{2\alpha }+{\left(\lambda \omega \right)}^{2}}$$

Changing λ to *τ*_0_ and α to *ν*. Equations (A29) and (A30) are the same Equations (13) and (14).
